# Performance Evaluation of a Novel Biosourced Co-Processed Excipient in Direct Compression and Drug Release

**DOI:** 10.3390/polym13060988

**Published:** 2021-03-23

**Authors:** Rihab Benabbas, Noelia M. Sanchez-Ballester, Adrien Aubert, Tahmer Sharkawi, Bernard Bataille, Ian Soulairol

**Affiliations:** 1 ICGM, University Montpellier, CNRS, ENSCM, 34090 Montpellier, France; noelia.sanchez-ballester@umontpellier.fr (N.M.S.-B.); adrien.aubert@umontpellier.fr (A.A.); tahmer.sharkawi@umontpellier.fr (T.S.); bernard.bataille@umontpellier.fr (B.B.); or ian.soulairol@chu-nimes.fr (I.S.); 2 Department of Pharmacy, Nîmes University Hospital, 30000 Nimes, France

**Keywords:** co-processed excipients, direct compression, alginic acid, melatonin

## Abstract

This study exposes the potential usefulness of a new co-processed excipient, composed of alginic acid and microcrystalline cellulose (Cop AA-MCC), for the preparation of immediate drug release tablets by direct compression. Evaluation of the physical and mechanical properties as well as the disintegration behavior of Cop AA-MCC in comparison to commercial co-processed excipients (Cellactose^®^, Ludipress^®^, Prosolv^®^ SMCC HD90 and Prosolv^®^ ODT) and to the physical mixture of the native excipients (MCC and AA), was carried out. The obtained results illustrate the good performance of Cop AA-MCC in terms of powder flowability, tablet tensile strength, compressibility, and disintegration time. Although, this new co-processed excipient showed a slightly high lubricant sensitivity, which was explained by its more plastic than fragmentary deformation behavior, it presented a low lubricant requirement due to the remarkably low ejection force observed during compression. Compression speed and dwell time seemed not to affect significantly the tabletability of Cop AA-MCC. The study exposed evenly the performance of Cop AA-MCC compared to Prosolv^®^ ODT, in terms of tabletability and dissolution rate of Melatonin. Cop AA-MCC presented comparable hardness, lower dilution potential, higher lubricant sensitivity, lower ejection force, and faster Melatonin’s release time than Prosolv^®^ ODT. In summary, Cop AA-MCC exhibited interesting physical, mechanical, and biopharmaceutical properties, which demonstrate its concurrence to commercially available co-processed excipients. Furthermore, the simplicity of its composition and the scalability of its elaboration makes this multifunctional excipient highly recommended for direct compression.

## 1. Introduction

Direct compression (DC) continues being the most preferred choice of the pharmaceutical industry for the production of compressed tablets. This manufacturing process represents a fast and simple method that provides an effective and successful tableting operation. It consists on preparing a simple physical mixture of an active pharmaceutical ingredient with the excipients and the lubricant, followed directly by their compression, without any additional processing steps [1,2].

The excipients used for DC process need to provide multiple functionalities, such as good powder flowability, high binding ability, low friction tendency and fast disintegration capacity, in order to perform an effective tablet production. These desirable requirements are difficult to find in a single material. In addition, the use of multiple excipients in the tablet formulation could lead to some heterogeneity problems (segregation) and incompatibility issues that can occur between the active pharmaceutical ingredient (API) and the different excipients used [3,4].

Nowadays, excipient manufacturers spend considerable efforts in the research and the development of new multifunctional excipients in order to overcome the above cited constrains and to suit as much as possible the active ingredients. Many research focus on excipient’s chemical modifications to create alternative functional direct compressible excipients. Alginate esters and cinnamyl-chitosan are recent examples of chemically modified compounds that have shown their effectiveness in direct compression and disintegration [5,6]. On the other hand, the most popular method being currently used for the development of novel materials presenting multiple functionalities is co-processing. This method consist of a physical combination of two or more existing excipients using an appropriate manufacturing process (spray drying, wet granulation, hot melt extrusion…) [7,8,9]. Commonly, the processed blends are mixtures of fillers, binders, and disintegrants aiming to produce a final compound, with better functional properties, intended to be used as excipient for direct compression. As a result and in the ideal case, the manufactured tablets would possess improved characteristics in terms of hardness, disintegration, lubricant sensitivity and API bioavailability, in addition to an enhancement of the powder flowability and bulk density [10,11].

Several, ready-to-use, co-processed excipients are already available on the market (Cellactose^®^, Avicel^®^ HFE, Prosolv^®^…) [10,12,13]. Although, they have shown their usefulness in several studies [10,12,14], the development of novel multifunctional excipients is still needed owing to the great number and variety of APIs.

In our previous work, the design, characterization, and optimization of a new co-processed excipient (Cop AA-MCC), based on alginic acid and microcrystalline cellulose, for the preparation of immediate drug release tablets by direct compression was reported. This study demonstrated clearly the effectiveness of the designed co-processed excipient in comparison to the native materials (MCC and AA) and to their physical mixture [15].

Cop AA-MCC represents an ideal excipient designed especially for direct compression. It is suited for the majority of directly compressible actives because it combines acceptable bulk density, good flow, and high compactibility. In addition, its considerable fast disintegration allows it to be formulated in orodispersible tablets and therefore to satisfy a large group of patients (including elderly and children).

Moreover, from a commercial point of view, Cop AA-MCC was produced only using two safe natural excipients (alginic acid and microcrystalline cellulose) that are largely available at a reasonable cost. The co-processing process used to obtain Cop AA-MCC was wet granulation; a classical technique, less expensive than spray drying and easily mastered by industrial staff. In addition, this industrial method allowed obtaining a good yield of the co-processed excipient.

To complete our previous study and to demonstrate further the potential usefulness of this new product, it was found essential to compare Cop AA-MCC to commercial co-processed excipients (Cellactose^®^, Ludipress^®^, Prosolv^®^ SMCC HD90, and Prosolv^®^ ODT) in order to see its concrete and realistic position in the current market. It was evenly compared to the physical mixture of the native excipients using two grades of MCC (Vivapur 101 and Vivapur 200) in formulation with AA. The main objective of this work was to perform a detailed evaluation of the performance of Cop AA-MCC in the preparation of fast-disintegrating tablets by direct compression. Therefore, the powder physical properties and the tablet mechanical behavior under different compression process parameters (compression speed, dwell time, and external and internal lubrication) as well as the tablet disintegration behavior of Cop AA-MCC in comparison to the above selected materials were investigated. Moreover, in order to test the suitability of this material in formulation, tablets containing an active pharmaceutical ingredient for immediate drug release were prepared. Thus, small tablets, suitable for pediatric dosage forms, were formulated using Melatonin as a drug model. The compaction study and the release profile were investigated for Cop AA-MCC and compared to Prosolv^®^ ODT. This latter was chosen because of its already proved effectiveness in the production of fast-disintegrating tablets by direct compression [14].

## 2. Materials and Methods

### 2.1. Materials

Alginic acid AA (Vivapharm^®^ Alginate PH 060, JRS PHARMA (Patterson, NY, USA)) and Microcrystalline cellulose MCC (Vivapur 101^®^, JRS PHARMA (Patterson, NY, USA)) were used for the preparation of Cop AA-MCC. Prosolv^®^ SMCC HD90, Prosolv^®^ ODT (JRS PHARMA, Patterson, NY, USA), Cellactose^®^ 80 (MEGGLE, Wasserburg, Germany), and Ludipress^®^ (BASF, Ludwigshafen, Germany) were used as excipients of reference. Two types of magnesium stearate (MgSt) were used as lubricant in this study: Ligamed MF-3-V (Peter Greven, Bad Münstereifel, Germany) for external lubrication and magnesium stearate (Baerlocher, Unterschleißheim, Germany) for internal lubrication.

Cop AA-MCC was obtained by wet granulation of a dry mixture composed of 10% AA (Vivapharm^®^ Alginate PH 060) and 90% MCC (Vivapur^®^ 101), as described in previous work [15].

Prosolv^®^ SMCC HD90 is a co-processed excipient composed of MCC and colloidal silicon dioxide. This grade is used as filler-binder in the formulation of pharmaceutical tablets, and it shows the best disintegration time compared to other Prosolv SMCC grades [16]. It was chosen as a reference excipient for Cop AA-MCC as it is mostly composed of MCC.

Prosolv^®^ ODT is a co-processed excipient, as well as Prosolv^®^ HD90, it is composed of silicified microcrystalline cellulose in addition to other excipients, which are fructose, mannitol and crospovidone. The crospovidone provides a faster disintegration allowing its use in the preparation of orodispersible tablets [17].

Cellactose^®^ 80 is a co-processed excipient obtained by spray drying of 75% α-lactose monohydrate and 25% of cellulose powder. It was chosen in the comparative with Cop AA-MCC because they are both composed of a ductile (cellulose) and a brittle material (lactose for Cellactose and AA for Cop AA-MCC) [12].

Ludipress^®^ is a multifunctional excipient that has been specially developed for direct compression. It is obtained by co-processing 93% of lactose monohydrate in combination with 3.5% of Kollidon^®^ 30 and 3.5% of Kollidon^®^ CL. In this study, this excipient is used to compare with Cop AA-MCC as a cellulose-free excipient [18].

Two dry mixtures, prepared from 10% of AA and 90% of MCC presenting different grades (Vivapur^®^ 101 for DM (which is the grade used for the preparation of Cop AA-MCC) and Vivapur^®^ 200 for DM2) were evenly used as references and compared to Cop AA-MCC.

### 2.2. Methods

#### 2.2.1. True Density

True density of the powders was measured using a helium pycnometer 1305 (Micromeritics, Norcross, GA, USA) and the required mass of powder for each measurement was about 3 g. Measurements were done in triplicate for each sample.

#### 2.2.2. Particle Size Distribution

The particle size distribution for all the powders was determined by dry laser diffraction (Mastersizer 2.18; Malvern Instruments Ltd, Malvern, United Kingdom). The powder samples were de-agglomerated with a pressure of 0.4 bars, and the feed rate was adjusted to 1.8. Each measure was performed at least in triplicate and the median particle diameter was used to express the particle size.

#### 2.2.3. Tapped and Bulk Density

Tapped (*ρT*) and bulk density (*ρB*) were measured by following the method described in the European Pharmacopoeia [19]. Their determination allowed calculating Carr’s index (*C*) and Hausner ratio (*H*) according to Equations (1) and (2), respectively. These two parameters expressed the flowability of the tested powders.

(1) C=100×(1−ρB/ρT)

(2) H=ρT/ρB.

#### 2.2.4. Angle of Repose

Powder flowability was also evaluated by the measurement of the angle of repose according to the European Pharmacopeia guidelines [20]. It was determined by allowing an excess quantity of each material (about 50 g) positioned above a fixed diameter base to drain from the container. Formation of a cone of powder on the fixed diameter base allowed determination of the drained angle of repose.

#### 2.2.5. Scanning Electronic Microscopy

Particle morphology was investigated by scanning electron microscopy (Hitachi 4800 S, Tokyo, Japan) after platinum sputtering under vacuum before observation.

#### 2.2.6. Compaction Study

##### Tabletability

Briefly, 500 mg of unlubricated powder of each excipient were compacted under different compaction pressures (100, 200, and 300 MPa) using a rotary tablet press simulator Styl’One Evolution (Medelpharm, Beynost, France). Flat round punches of 11.28 mm diameter were used for the compaction study, and the speed of punches was of 9.5 mm/s (15%).

Tablet thickness, diameter, and hardness were measured using a Sotax MultiTest 50FT (Sotax AG, Basel, Switzerland). Measurements were carried out on 5 tablets per compaction point, and results were expressed as the mean value ± standard deviation.

The diametric tensile strength (*T_S_*) that expresses accurately the powder tabletability was calculated from the crushing force using Equation (3)

(3) Ts=2 FπDh,

where *F* is the diametric force necessary to break the cylindrical compact, *D* is the diameter of the compact, and *h* is its thickness.

##### Compressibility

Powders’ compressibility was evaluated by the measurement of tablet porosity according to Equation (4)

(4) ε=1−ρr.

In which the relative porosity (*ρr*) is calculated according to Equations (5) and (6)

(5) ρr=ρTa/ρTr

(6) ρr=mT/πR2hTρTr,

where *ρTa* is the density of tablet, *ρTr* true density, *R* the radius of tablet, *hT* its height, and *mT* its mass.

##### Elastic Recovery

The elastic recovery (*ER*) of each material was calculated using the Analis^®^ software (Medelpharm, Beynost, France). It corresponds to the evolution of the tablet thickness between the compression peak and the measurement of the thickness outside the matrix, immediately after compression. This evolution is characterized by percentage (%). The measurement was performed according to Equation (7)

(7) $$ER=\frac{Th-D}{D}\cdot 100,$$

where *D* is the distance between the punches during the compression peak and *Th* is the measured thickness of the tablet outside of the matrix.

##### Walker and Heckel Modeling

For Heckel modeling, three tablets of the different materials, compressed at 200 MPa, were used. They presented a constant mass of 500 mg. True densities (*ρ*) measured previously with the helium pycnometer were used to calculate Heckel mean yield pressures (Py), which are given by the inverse values of the slope of Equation (8)

(8) $$Ln\left(\frac{1}{1-D}\right)=KP+A.$$

K is the slope of the linear part of the plot (with the best R^2^ fit). A is the Y axis intercept with the linear part of the Heckel plot. Hersey and Rees [21] considered that Py values can be used to characterize the deformation mechanism of materials. The low value of Py reflects the plastic deformation of a hard-ductile powder, while Py high value reflects a fragmentary deformation of a brittle-soft material under compaction force (very hard materials possess Py value lower than 40 MPa, while very brittle materials have a Py value higher than 200 MPa).

For Walker modeling, true density is also used to determine the evolution of the powder relative volume with the increase in the compaction pressure. Walker [22] defines «W» as the compressibility coefficient, which represents the slope of Equation (9)

(9) 100V=−W∗log(p)+C,

where *V* is the relative volume, *P* is the compaction pressure, and *C* is the constant. The compressibility coefficient W indicates a measure of the irreversible compressibility of the compact, the higher is the W value, and the better is the compressibility of the powder. This model is more robust and more repeatable than Heckel but less accurate.

##### Ejection Force

The ejection force represents the maximum effort for ejecting the tablet out of the matrix. It was determined automatically by the compression simulator for the seven materials tested in the pure form (without lubricant).

##### Lubricant Sensitivity

Materials’ lubricant sensitivity was investigated by mixing them, for 5 min, with two different concentrations of magnesium stearate (0.5% and 2.5%), using a 3D mixer (Turbula^®^), in order to evaluate the effect of the internal lubrication on tablet tensile strength and disintegration time compared to tablets obtained without lubricant.

External lubrication was evenly carried out using an external lubrication system (Medel’Pharm, France). The lubricant is conveyed by compressed air and filled into a narrow tube in the supply part. The «puff» is generated by the Venturi inside the air blow cabinet (5–6 bars). The microdosing unit can contain a maximum of 4 g of lubricant. The amount of the lubricant pulverized can be adjusted by the setting of the position (high or low) of the container and the lubrication time can be fixed by the Analis software. The punches and the die walls are lubricated at the same time. For this study, the lubrication time tested was 500 ms with the amount of lubricant pulverized set in high position.

##### Speed and Dwell Time Effect

The effect of compression speed and dwell time on the tablet tensile strength was studied by producing tablets at different machine speeds (15%, 25%, and 50%) and dwell times (0, 500, and 1000 ms).

#### 2.2.7. Disintegration Time

The disintegration test was performed according to the method described in the European Pharmacopeia guidelines, disintegration of tablets and capsules monograph [23] using a disintegration apparatus Sotax DT50 (Sotax AG, Basel, Switzerland). Six tablets of each formulation were tested simultaneously and the results expressed as the mean value ± standard deviation. The end point was achieved when no residues were present on the bottom of the test basket.

#### 2.2.8. Melatonin Tablets Manufacturing

To explore the interest of Cop AA-MCC as a multifunctional excipient, suitable for direct compression, it was tested in the preparation of immediate drug release tablets. Prosolv^®^ ODT was chosen as a reference commercial excipient because it contains MCC on its composition, and it presents a very fast disintegration [14].

For this purpose, four different formulations containing one of the following co-processed excipients (Cop AA-MCC or Prosolv ODT) and 10% of melatonin as an active pharmaceutical drug model, with or without lubricant (0.5% of MgSt) (CM: Cop AA-MCC/Melatonin, PM: Prosolv ODT/Melatonin, CMM: CM/MgSt, PMM: PM/MgSt), were prepared. The different formulations were mixed using a 3D mixer (Turbula^®^) for 10 min, and compressed at 5 kN using the above-described tablet compression simulator (Styl’One^®^ Evolution). Concave round punches of 5.5 mm of diameter were used, at a speed of 9.5 mm/s (15%), in order to prepare tablets of 50 mg, dosed with 5 mg of melatonin. The obtained tablets were evaluated regarding their tensile strength, disintegration time, ejection force, and dissolution profile. Equation (10) was used to determine the tensile strength for convex tablets

(10) $$Ts=\frac{10F}{\pi \;D^{2}}\;{\left(2.84\frac{h}{D}-0.126\frac{h}{w}+3.15\frac{w}{D}\right)}^{-1},$$

where *F* is the breaking force, *D* is the diameter of the tablet, *h* is the thickness of the tablet, and *w* is the thickness of the central cylinder.

#### 2.2.9. Dissolution Profile

Dissolution tests were carried out using a paddle dissolution apparatus (Pharmatest DT70; Pharmatest, Hainburg, Germany), which is referred to as Apparatus 2 in the European Pharmacopeia [24]. For this 0.1 N Hydrochloric acid (500 mL) was used as dissolution medium, at 37 °C and under a rotational paddle-speed of 100 rpm. Samples were analyzed automatically each 1 min using a continuous flow-through system attached to an 8 cell UV/VIS spectrophotometer (Specord 250, Analytik Jena, Jena, Germany) at a wavelength of 223 nm for melatonin absorption. For each tablet formulation, all experiments were performed in triplicates and results were expressed as mean value with standard deviation.

## 3. Results and Discussion

### 3.1. Study of the Supplied Materials

#### 3.1.1. True, Bulk and Tapped Density

As presented in Table 1, Cop AA-MCC possesses a true density value (1.49 g/cm^3^) similar to the other tested materials. While Prosolv HD90 has shown the highest value of true density (1.53 g/cm^3^), DM2 has shown the lowest (1.45 g/cm^3^). The true density value is important to express powder compressibility, tablet porosity, and deformation behavior [25,26]. It is also considered as a critical property that can have a potential impact on mechanical product attributes during compression [27]. Regarding bulk density results, Cop AA-MCC presented lower values than the commercial materials and slightly higher than the dry mixtures. The feeble improvement on the bulk density of Cop AA-MCC compared to the dry mixture DM (which corresponds to the physical mixture of the primary materials: AA and MCC 101) can be attributed to the densification of the particles during the co-processing by wet granulation process [28]. This observation suggests that a potential enhancement in the powder flowability and hence in the die filling during the direct compression process would be obtained [29,30].

#### 3.1.2. Scanning Electronic Microscopy

**Figure 1:**
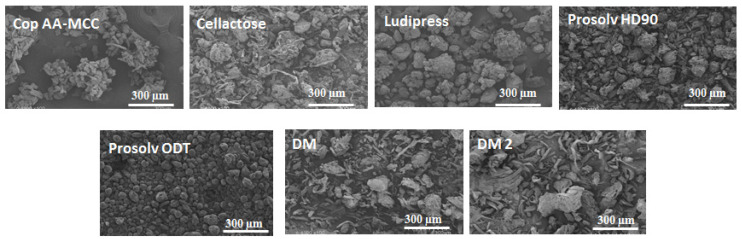
SEM images of the different co-processed excipients (Cop AA-MCC, Cellactose, Ludipress, Prosolv HD90, and Prosolv ODT) and the dry mixtures (DM and DM2).

As observed from SEM results (Figure 1), Cop AA-MCC powder consists of more or less spherical granules, obtained from particles’ agglomeration. Cellactose is composed of heterogeneous shapes’ particles resulting from the spray drying process; some are granular, whereas others are fibrous. Ludipress particles are spherical with a rocky appearance and rough surfaces. DM and DM2 powders are composed of two types of particles: MCC particles (granular and/or irregular form) and AA particles (elongated particles). Finally, Prosolv HD90 particles are almost completely granular, whereas Prosolv ODT particles are mostly spherical.

Regarding the particle size, the studied materials have particles of different sizes. SEM results show that Cop AA-MCC particle size is more comparable to Cellactose and Ludipress rather than to the other materials. These observations were confirmed by the laser diffraction results (Table 2).

Thus, from the SEM images, it can be concluded that the materials tested possess different morphologies. This is an influencing factor, which can largely affect powders’ flow properties, and their compaction behavior [27].

#### 3.1.3. Particle Size and Powder Flowability

Particle size and powders flowability results are expressed in Table 2.

As shown in Table 2, Ludipress was found to possess the best flowability, which is associated to its large particle size and rounded shape [2]. Then, Cop AA-MCC and Prosolv HD 90 were positioned in second place as they presented a good to fair powder flow. For Cop AA-MCC, this is attributed to its larger particles’ size with more or less granular shape. On the other hand, for Prosolv HD90, the good flow comes from the presence of colloidal silica. The mechanism behind this enhancement is based on inter-particle forces disruption by silica particles adhered to MCC particles’ surfaces [31].

Prosolv ODT was found to possess a fair flow due to the presence into its composition of constituents presenting particles with various sizes. As well as Prosolv ODT, Cellactose presented a fair flow, related to the spherical and some fibrous particle’s shape obtained by spray drying. Finally, the dry mixtures (DM and DM2) presented both passable to poor flow properties despite the larger particle’ size of DM2 compared to DM. This is explained by the presence in both powders of elongated AA particles, which lead to a poor flow and to the sequestration of void spaces in the powder bed [2,29,30].

In terms of flowability, Cop AA-MCC seems to be interesting for direct compression applications. This is supported by the results obtained from the comparative analysis realized with commercial co-processed excipients.

#### 3.1.4. Powders’ Tabletability, Compressibility and Elastic Recovery of Non-Lubricated Materials

**Figure 2:**
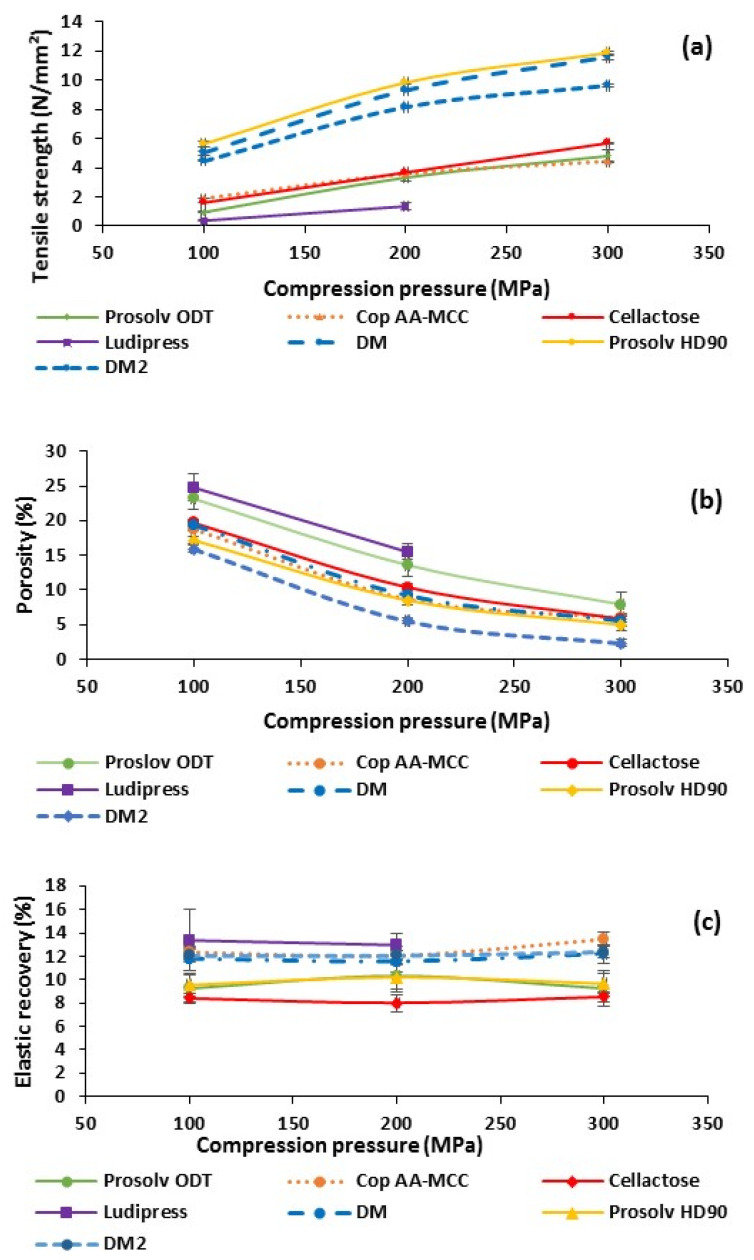
Tablets’ tensile strength (**a**), porosity (**b**), and elastic recovery (**c**) as function of compression pressure of all the non-lubricated tested materials.

As shown in Figure 2a, pure Cop AA-MCC tablets exhibit good tensile strength values ranging from 1.61 to 5.70 N/mm^2^ at different compaction pressures. Similar hardness was observed for tablets obtained with Cellactose and Prosolv ODT. On the other hand, Prosolv HD 90, DM, and DM2 presented the highest tablet’s tensile strength as they are mostly composed of MCC, which possesses a plastic powder deformability under compaction pressure and a high particle–particle binding capacity [32,33]. Moreover, the presence of colloidal silica in Prosolv HD90 was found to improve further the powder compactibility of MCC [13].

The higher tabletability showed by DM compared to DM2 is explained by the differences in their particle sizes. In fact, DM is composed of smaller particles, which can strongly bound together after compression as a result of their high specific surface area. In this item, various studies have reported the relationship between powder particle size and tablet mechanical properties, mainly tensile strength [34].

Ludipress showed the lowest tabletability at both 100 and 200 MPa and no tablets could be obtained at 300 MPa. This co-processed excipient is composed of a large amount of lactose, which has a fragmentary powder deformation behavior [35] and a weak binding ability, resulting in the formation of weak compacts [36]. In addition, all the tablets obtained from Ludipress underwent surface and peripheral fractions and irregularities (chipping) during ejection, which weakened them even more. These observations are due to the sticking and adhesion of Ludipress powder to the machine tools as a result of frictions and radial elastic recovery during ejection [2]. In fact, these defects are the reason behind the impossibility of preparing pure tablets of Ludipress at 300 MPa. Hence, Ludipress powder lubrication is required before direct compression.

Along the same lines, the analysis of compressibility results, showed in Figure 2b, revealed that DM2 presented the lowest powder porosity. Cop AA-MCC, Cellactose, Prosolv HD90, and DM presented almost identical powder porosities, which decreased further with the increase in the compaction pressure. This is explained by a good particle arrangement and packing characteristics, under compressive force, of the different abovementioned excipients [30,37]. Ludipress and Prosolv ODT showed lower compressibility’s values as they presented slightly higher tablet porosities. This could be related to the complex composition of those co-processed excipients, leading to a worse particle arrangement during compression.

Finally, for the elasticity results (Figure 2c), Cop AA-MCC was found to be less elastic than Ludipress, as elastic as DM and DM2 and more elastic than Prosolv ODT, Prosolv HD90, and Cellactose. Its elasticity is mainly correlated to the presence of AA particles in the granules’ surfaces, which were previously found to possess a high elastic recovery [15,38].

From a general view, all the excipients presented relatively low elastic recovery results, confined between 8% and 16%, suggesting their low elastic behavior. The higher elasticity observed for Ludipress could be probably the reason behind the defects (chipping) previously observed during ejection of its tablets [2].

#### 3.1.5. Ejection Force of Non-Lubricated Materials

**Figure 3:**
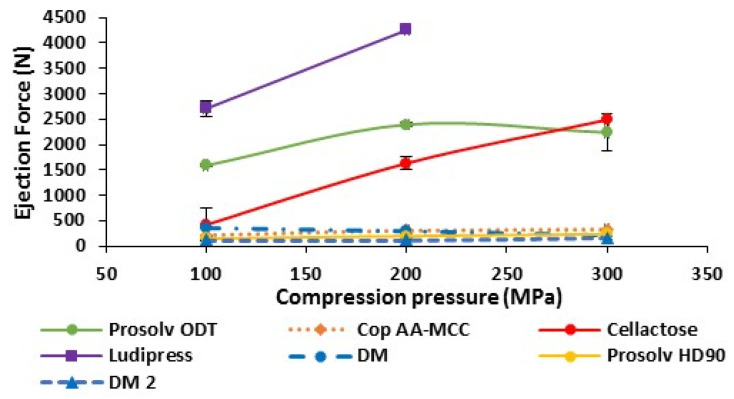
Tablet’s ejection force at different compaction pressures of non-lubricated materials.

As illustrated in Figure 3, Cop AA-MCC, Prosolv HD90, DM, and DM2 presented similar, low tablet ejection forces when they were tested without lubricant in direct compression. This is attributed to the presence of large amount of MCC in the composition of all those materials. The MCC’s low friction behavior during ejection has already been described in the literature [39].

To note that this is highly advantageous for an effective tablet manufacturing process. The low ejection force indicates the low tendency of a powder to friction and sticking. Such type of powders does not generally need lubrication because they exhibit a plastic deformation behavior under compaction pressure. Thus, less new surfaces are created and less adhesion to tooling occurs during compaction process [39,40]. On the other hand, Cellactose, Prosolv ODT, and mainly Ludipress, presented high ejection forces, which increased with the increase in the compaction force. This type of behavior is associated to fragmentary deforming materials. With those materials, more surfaces are created during the compaction process, leading to more pronounced sticking and adhesion phenomena. Therefore, the use of lubricant in mixtures with those excipients is required before compression in order to reduce their adhesion to tooling and to decrease the particles’ frictions.

#### 3.1.6. Powder Deformation Behavior of Non-Lubricated Materials

**Figure 4:**
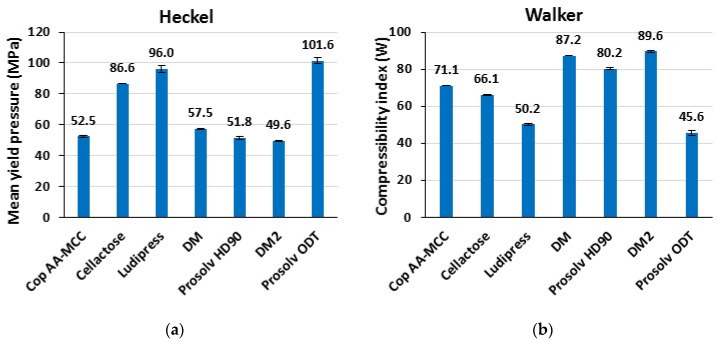
(**a**) Heckel and (**b**) Walker modeling for powder deformation.

Heckel and Walker modeling were used as approximate indicators to compare powders’ deformation behavior. Figure 4 shows the Py and the W results for the different studied materials. An excipient is considered to be more plastic when it possesses the smallest value of Py and the biggest value of W [36]. Prosolv HD90, DM, and DM 2 were found to be the most plastic materials, followed by Cop AA-MCC, then Cellactose, Ludipress, and finally Prosolv ODT. This is in agreement with previous reported literature as Prosolv HD90, DM, and DM2 are mainly constituted of MCC (which is a ductile material possessing a plastic deformation mechanism) [33,39]. While Cellactose and Prosolv ODT are composed, in addition to MCC, of other ingredients that are less ductile than MCC. On the other hand, Ludipress (Py > 80 MPa) is almost completely formed of lactose (93%) which is a brittle material that undergoes a fragmentary deformation mechanism under compaction pressure [33,41].

Moreover, the analysis of Cop AA-MCC by Heckel and Walker modeling reveals its more plastic than brittle deformation behavior (less plastic than Prosolv HD90, DM, and DM2 and more plastic than Cellactose, Prosolv ODT, and Ludipress). The observed loss in plasticity compared to the dry mixtures may be attributed to the hornification phenomena, as a result of the humidifying and drying steps occurred during the co-processing by wet granulation as was discussed in previous work [42]. In general, the deformation behavior results are in agreement with the previous ejection force results (Figure 3).

#### 3.1.7. Disintegration Time of Non-Lubricated Materials

**Figure 5:**
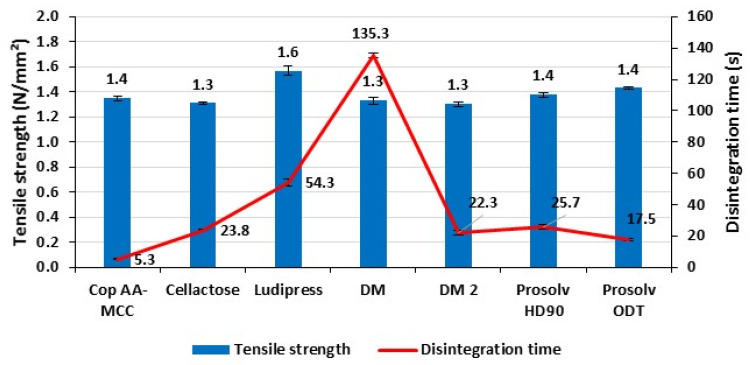
Disintegration time of the non-lubricated tested materials at a tensile strength confined between 1.3 and 1.6 N/mm^2^.

Figure 5 shows the disintegration times of non-lubricated tablets (500 mg) obtained from the different tested materials and possessing comparable tensile strength confined between 1.3 and 1.6 N/mm^2^. It is interesting to notice that although possessing the highest tensile strength among the tested materials, Cop AA-MCC tablets presented the fastest disintegration time (5–6 s), followed by Prosolv ODT, DM2, Cellactose, Prosolv HD90, Ludipress, and lastly DM.

Disintegration times obtained for DM and DM2 clearly showed the effect of the different particle size of the microcrystalline cellulose used in each mixture [43]. DM possesses the smaller particle size disintegrated slower than DM2. This is explained by the fact that smaller particles create higher bonding surfaces than larger particles. In fact, the tablets produced with smaller particles would need greater degree of solvation to break these bonds, and thus more time to achieve their complete disintegration [43].

Disintegration results suggest that Cop AA-MCC can be an effective competitor to commercial co-processed materials in terms of fast disintegration. This property is obtained from a combination of capillary and swelling action, owing the presence of both AA and MCC in the granule structure, as it was found previously [15].

The comparison between the tested commercially available materials shows that Prosolv ODT presented the shortest disintegration time and the closest one to Cop AA-MCC.

#### 3.1.8. Effect of Compression Speed and Dwell Time on Tablet Tensile Strength

**Figure 6:**
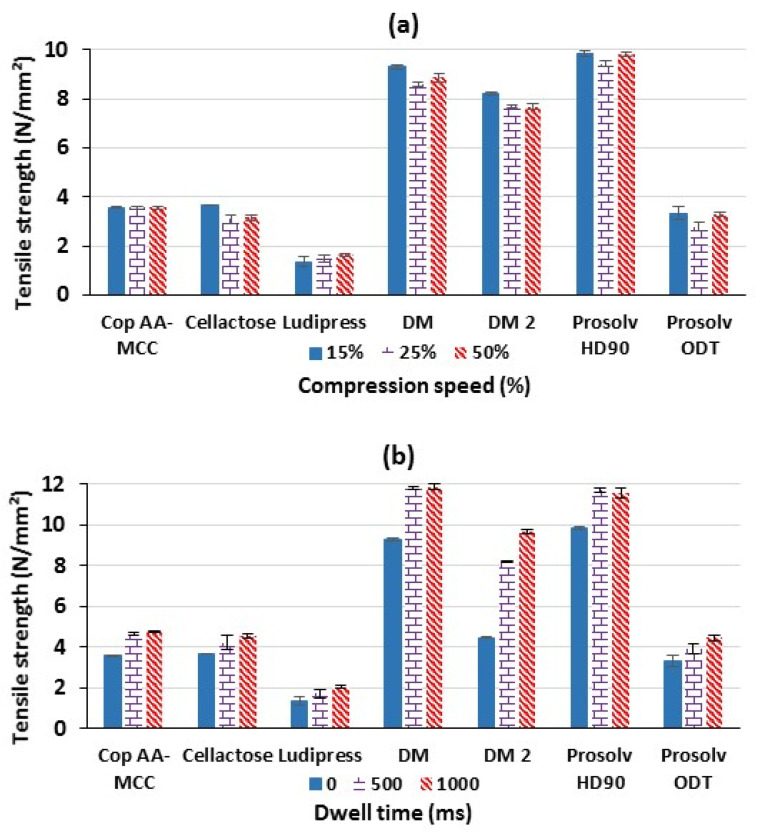
Effect of (**a**) compression speed and (**b**) dwell time on tablet’s tensile strength, at 200 MPa.

According to previous reports, generally an increase in the compression speed leads to a decrease in tablets’ tensile strength as a result of the reduced duration of particle–particle bond formation [44]. However, it was deduced from Figure 6a that an increase in the compression speed affected very slightly the tensile strength of the tablets obtained from the tested materials. Interestingly, Cop AA-MCC was almost not affected by the variation on the compression speed, while only Cellactose tablets showed the highest speed sensitivity.

Tablet irregularities (capping, sticking, and chipping) could also occur when increasing the compression speed due to the generation of stronger friction forces. While this was observed for Ludipress, Prosolv ODT, and Cellactose, no defects have been observed for Cop AA-MCC, DM, DM2, and Prosolv HD90 for all compression speeds tested.

On the other hand, the analysis of the dwell time, which corresponds to the duration of maintaining the powder bed between the two punches at the maximal compression force, revealed that an increase in this parameter affected positively the tablet’s tensile strength of all materials (Figure 6b). This is related to an enhancement in the duration of the particles’ plastic and/or fragmentary deformation. Thus, the time of particle–particle adhesion is prolonged, at the maximal force of compression [45]. Especially, at 500 ms of dwell time, the tensile strength of the tablets produced from the different materials was markedly affected. However, a further raise in the tablet dwell time (1000 ms) for Cop AA-MCC, Prosolv HD90, and DM had no additional effect on the tensile strength, suggesting that the maximum particle–particle bonding was already achieved for all those powders at 500 ms. Contrariwise, DM2, Prosolv ODT, Ludipress, and Cellactose kept increasing their tensile strength at higher dwell time (Supplementary Table S1).

### 3.2. Study of Lubricated Materials

#### 3.2.1. Effect of Lubrication on Tablet Tensile Strength

**Figure 7:**
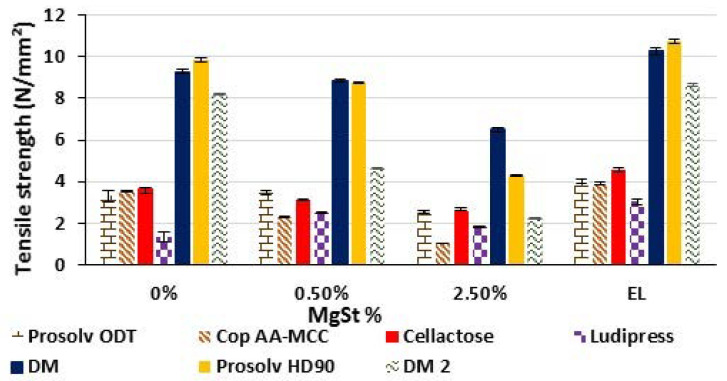
Effect of internal lubricant concentration and external lubrication (EL) on tablet tensile strength of the different studied materials.

Figure 7 shows the influence of external (EL) and internal lubrication (using 0, 0.5, and 2.5% of MgSt) on the tensile strength of tablets produced from the different studied materials.

In general, a material’s dependent behavior was observed for internal lubrication. A decrease in tablets’ tensile strength with the increase in MgSt % in the formulation (from 0% to 2.5%) was markedly observed for Cop AA-MCC, Prosolv HD90, DM, and DM2, whereas Cellactose was very slightly affected. Generally, the excipient lubricant sensitivity is correlated to its particle deformation [46,47]. The more plastic the material deformation is, the more it is influenced by the lubrication. With such materials, the presence of lubricant prevents the particle–particle adhesion while with brittle materials, new surfaces are always being created during compression and thus the binding ability is almost not affected [40].

On the contrary, Ludipress and Prosolv ODT benefited from the addition of 0.5% lubricant. Their tablet tensile strength increased owing to the reduction in sticking previously observed for pure tablets. However, the addition of a higher lubricant concentration (2.5%) caused a reduction in their tensile strength.

For external lubrication (EL), it was observed that the deposition of the lubricant on the surface of the die cavity and the two punches, using the external lubrication device, has an increasing effect on the tablet tensile strength for all tested materials [48,49]. This is explained by the reduction in tablet irregularities (fractures and chipping) as a result of the decrease in tablet ejection forces, which was obtained due to a diminution of friction and adhesion of the powder to the machine’s tooling. Furthermore, using the external lubrication method, the lubricant particles do not interact with the excipient particles in the powder bed. Thus, no alteration on the particle–particle adhesion and bond formation would occur [48,49].

Briefly, the tablet tensile strength sensitivity to the internal lubrication is mainly correlated to the material’s deformation behavior [46,48].

#### 3.2.2. Effect of Lubrication on Tablet’s Disintegration

**Figure 8:**
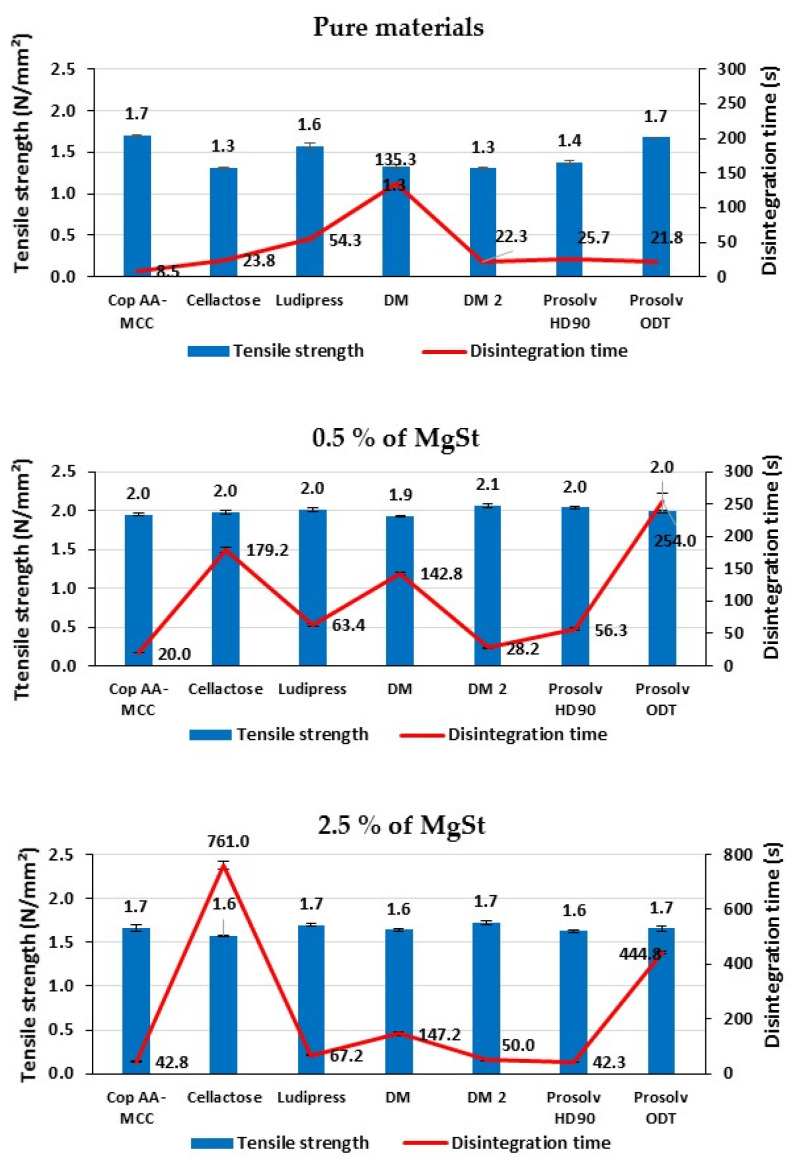

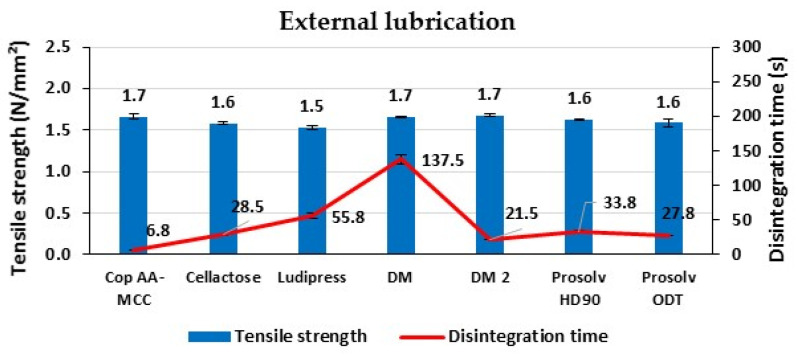
Effect of internal lubricant concentration and external lubrication (EL) on tablet disintegration time of the different studied materials.

The impact of internal and external lubrication on tablet’s disintegration time, evaluated at nearly identical tablet tensile strength, is shown in Figure 8. In general, internal lubrication results show a difference in the disintegration sensitivity to lubrication between the tested materials. As was observed previously for pure materials, Cop AA-MCC presented the fastest disintegration among all the tested materials. At 0.5% MgSt, an increase in tablet’s disintegration time was significantly observed for Cellactose and Prosolv ODT due to the hydrophobic character of MgSt [14], while Ludipress, Cop AA-MCC, DM, and DM2 showed low sensitivity to lubrication. Prosolv HD 90 was moderately influenced. The fast disintegration observed with Prosolv HD90, DM, and DM2 is not only correlated to their functional properties (swelling and/or capillary action) but also to tablet’s porosity (about 34–38% vs. 20–22% for the other studied materials), at the tested tablet tensile strength (~1.30 N/mm^2^). For Cop AA-MCC, the fast disintegration is related to its disintegration mechanism, which combines capillarity and swelling [15,50,51], making it less sensitive to the hydrophobicity of MgSt and comparable to sodium starch glycolate [51]. At 2.5% MgSt, similar and clearer behaviors were observed than for 0.5% MgSt, for all the studied materials.

On the other hand, the external lubrication (EL) has almost not delayed the disintegration time for all the tested materials compared to pure tablets. Using this type of lubrication, the lubricant is pulverized rapidly onto the punches and the die walls. Only an external thin layer of lubricant is formed on the tablet surface and the possibility of distribution of MgSt within the tablets seems negligible thus retarding very slightly the tablets’ disintegration contrary to the internal lubrication where MgSt particles are included in the powder bed [49].

It is interesting to conclude that Cop AA-MCC was found to present a short disintegration time, compared to the other co-processed excipients, whatever the lubrication process and the lubricant concentration used.

### 3.3. Study of a Melatonin Tablets

#### 3.3.1. Compaction Study

Cop AA-MCC was tested as a multifunctional excipient in the preparation of small tablets (50 mg) for immediate drug release compared to Prosolv^®^ ODT. This commercial excipient was chosen as a reference because it contains MCC on its composition [17] and presented similar tablet hardness and fast disintegration as Cop AA-MCC (Figure 2 and Figure 4) [14].

**Figure 9:**
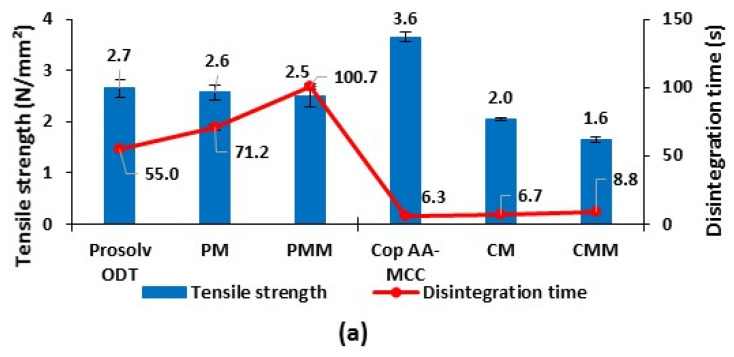

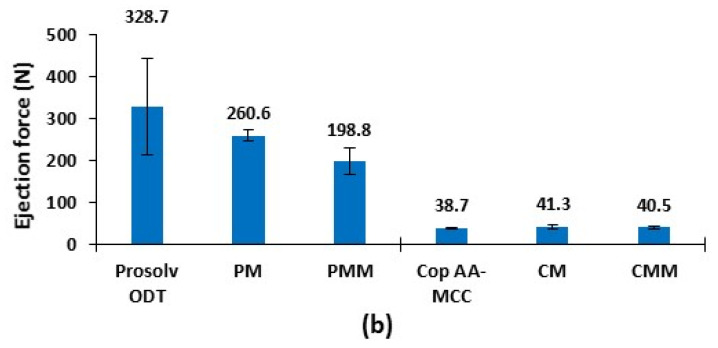
(**a**) Tensile strength, disintegration time, and (**b**) ejection force of tablets obtained from Cop AA-MCC, Prosolv ODT, CM: Cop AA-MCC/Melatonin, PM: Prosolv ODT/Melatonin, CMM: CM/MgSt, and PMM: PM/MgSt, compressed at 5 kN.

As shown in Figure 9a, tensile strengths of tablets prepared from pure co-processed excipients at 5 kN are relatively high, especially for Cop AA-MCC (2.7 N/mm^2^; 3.6 N/mm^2^ for Prosolv ODT (PM) and Cop AA-MCC (CM), respectively). The addition of 10% of melatonin reduced the tensile strength of both excipients but this effect was more notable for Cop AA-MCC (2.6 and 2.0 N/mm^2^ for PM and CM, respectively), which is explained by the poor tabletability of melatonin. It can also be deduced from this observation that Prosolv ODT has better dilution potential [52] than Cop AA-MCC. Along the same lines, the addition of 0.5% MgSt further reduced the tensile strength and more significantly, for Cop AA-MCC, as was found previously.

Regarding the disintegration time, Cop AA-MCC tablets disintegrated faster than Prosolv ODT tablets, whatever was their composition (pure or formulated). The first one disintegrated by the swelling and the effective water sorption of both AA and MCC [15,50,51]. The second one disintegrated mainly by the wicking and the recovery of elastic energy of crospovidone [53,54].

For the ejection force results, shown in Figure 9b, pure and formulated Prosolv ODT tablets presented higher ejection forces than Cop AA-MCC tablets. This is due to the high sticking and adhesion of Prosolv ODT powder to the punches and to the die walls. At 0.5% MgSt, a reduction in its ejection force was clearly observed. On the other hand, Cop AA-MCC tablets presented low ejection force values regardless their composition (with or without lubricant). The addition of MgSt had not a significant effect on the reduction in tablet ejection force for Cop AA-MCC. These results showed that this latter has no lubricant requirement due to its high low friction’s tendency due to its more plastic character compared to Prosolv ODT.

#### 3.3.2. Tablets Dissolution Profile

**Figure 10:**
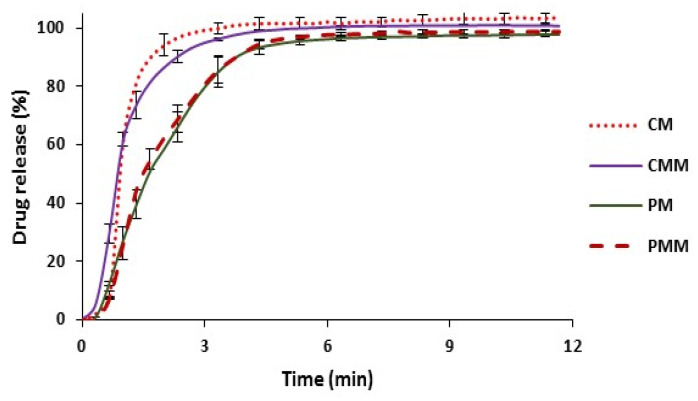
Dissolution profiles of CM (Cop AA-MCC/Melatonin), PM (Prosolv ODT/Melatonin), CMM (CM/MgSt), and PMM (PM/MgSt) tablets compressed at 5 kN.

Dissolution profiles of the prepared tablets are illustrated in Figure 10. It was noticed that Cop AA-MCC tablets showed faster API dissolution profile than Prosolv ODT tablets, which is coherent with disintegration results. It is interesting to note, that the addition of hydrophobic MgSt did not significantly affect the dissolution rate of both tested formulations.

## 4. Conclusions

The present work deals with the evaluation in direct compression of the functionality of a new co-processed excipient (Cop AA-MCC) in comparison to commercial co-processed excipients. The obtained results showed that Cop AA-MCC possesses a fair flowability, a relatively high tensile strength, and a very rapid tablet disintegration. Besides, it presented no lubricant requirement due to its extremely low ejection force and friction’s tendency, at different compaction pressures, allowing it to overcome its mechanical sensitivity to lubrication. Moreover, its tablets’ tensile strength has shown low sensitivity to the variation of dwell time and compression speed, which is recommended for a successful compression operation. Finally, formulation of Cop AA-MCC with melatonin has proven a more rapid dissolution rate compared to the formulation of melatonin with Prosolv^®^ ODT. Thus, it can be concluded that Cop AA-MCC is an interesting biosourced co-processed excipient that possesses a great potential to be explored and exploited industrially.

## Figures and Tables

**Table 1:** True (TRD), bulk (BD), and tapped (TPD) density of the different tested materials.

Excipients	Cop AA-MCC	Cellactose	Ludipress	DM	DM2	Prosolv HD90	Prosolv ODT
TRD * (g/mL)	1.49	1.47	1.46	1.51	1.45	1.53	1.51
BD * (g/mL)	0.36	0.41	0.55	0.33	0.35	0.47	0.60
TPD * (g/mL)	0.43	0.49	0.64	0.44	0.46	0.56	0.74

* SD ≤ 0.02.

**Table 2:** Particle size and powder flowability expressed by Carr’s index, Hausner ratio, and angle of repose.

	Cop AA-MCC	Cellactose	Ludipress	DM	DM2	Prosolv HD90	Prosolv ODT
PS (µm)	209 ± 1	197 ± 4	220 ± 13	86 ± 7	280 ± 5	151 ± 1	160 ± 5
AOR (°) *	37.9	38.2	30.1	47.1	42.4	35.8	37.9
CI *	15.5	18.0	14.3	24.9	22.8	15.9	19.7
HR **	1.18	1.22	1.17	1.33	1.29	1.19	1.24
Flow property	Good/fair	Fair	Good	Poor	Passable	Good/fair	Fair

* SD ≤ 0.5, ** SD ≤ 0.01 PS: particle size; AOR: angle of repose; CI: Carr’s index; HR: Hausner ratio.

## Data Availability

The data presented in this study are available on request from the corresponding author.

## Supplementary Materials

The following are available online at https://www.mdpi.com/2073-4360/13/6/988/s1, Table S1: Effect of compression speed and dwell time on tablet’s tensile strength, at 200 MPa.
