# Supplementary material for: Performance Evaluation of a Novel Biosourced Co-Processed Excipient in Direct Compression and Drug Release
Source: Polymers (Basel). 2021 Mar 23;13(6):988. doi: 10.3390/polym13060988 (PMC8004800; doi:10.3390/polym13060988)
Supplement: Supplementary file 1 [file polymers-13-00988-s001.pdf]

Table S1. Effect of compression speed and dwell time on tablet's tensile strength, at 200MPa.

Excipients tensile strength (MPa)	Dwell time (ms)			Compression speed (%)		
	0	500	1000	15	25	50
**Cop AA-MCC**	3.6 ± 0.0	4.6 ± 0.1	4.7 ± 0.1	3.6 ± 0.0	3.6 ± 0.1	3.6 ± 0.0
**Cellactose**	3.7 ± 0.0	4.2 ± 0.4	4.6 ± 0.1	3.7 ± 0.0	3.1 ± 0.2	3.2 ± 0.1
**Ludipress**	1.4 ± 0.2	1.7 ± 0.2	2.1 ± 0.1	1.4 ± 0.2	1.5 ± 0.2	1.7 ± 0.1
**DM**	9.3 ± 0.1	11.8 ± 0.1	11.9 ± 0.2	9.3 ± 0.1	8.6 ± 0.1	8.9 ± 0.2
**DM 2**	4.5 ± 0.0	8.2 ± 0.1	9.7 ± 0.1	8.2 ± 0.1	7.7 ± 0.1	7.7 ± 0.1
**Prosolv HD90**	9.8 ± 0.1	11.7 ± 0.1	11.6 ± 0.2	9.8 ± 0.1	9.4 ± 0.1	9.8 ± 0.1
**Prosolv ODT**	3.3 ± 0.3	3.9 ± 0.3	4.5 ± 0.1	3.3 ± 0.3	2.8 ± 0.2	3.3 ± 0.1
